# The ACCESS study a Zelen randomised controlled trial of a treatment package including problem solving therapy compared to treatment as usual in people who present to hospital after self-harm: study protocol for a randomised controlled trial

**DOI:** 10.1186/1745-6215-12-135

**Published:** 2011-05-26

**Authors:** Simon Hatcher, Cynthia Sharon, Allan House, Sunny Collings, Varsha Parag, Nicola Collins

**Affiliations:** 1 Department of Psychological Medicine, Faculty of Medical and Health Sciences, The University of Auckland, Private bag 92019, Auckland, New Zealand; 2 Leeds Institute of Health Sciences, The University of Leeds, Leeds, UK; 3 Social Psychiatry & Population Mental Health Research Unit, University of Otago, Wellington, New Zealand; 4 Clinical Trials Research Unit, School of Population Health, The University of Auckland, New Zealand

## Abstract

**Background:**

People who present to hospital after intentionally harming themselves pose a common and important problem. Previous reviews of interventions have been inconclusive as existing trials have been under powered and done on unrepresentative populations. These reviews have however indicated that problem solving therapy and regular written communications after the self-harm attempt may be an effective treatment. This protocol describes a large pragmatic trial of a package of measures which include problem solving therapy, regular written communication, patient support, cultural assessment, improved access to primary care and a risk management strategy in people who present to hospital after self-harm using a novel design.

**Methods:**

We propose to use a double consent Zelen design where participants are randomised prior to giving consent to enrol a large representative cohort of patients. The main outcome will be hospital attendance following repetition of self-harm, in the 12 months after recruitment with secondary outcomes of self reported self-harm, hopelessness, anxiety, depression, quality of life, social function and hospital use at three months and one year.

**Discussion:**

A strength of the study is that it is a pragmatic trial which aims to recruit large numbers and does not exclude people if English is not their first language. A potential limitation is the analysis of the results which is complex and may underestimate any effect if a large number of people refuse their consent in the group randomised to problem solving therapy as they will effectively cross over to the treatment as usual group. However the primary analysis is a true intention to treat analysis of everyone randomised which includes both those who consent and do not consent to participate in the study. This provides information about how the intervention will work in practice in a representative population which is a major advance in this study compared to what has been done before.

**Trial registration:**

Australia and New Zealand Clinical Trials Register (ANZCTR): ACTRN12609000641291

## Background

Hospital attendance following self-harm is important because it is common, and it is a risk for subsequent suicide and for increased mortality from all causes. In 2006 there were 5400 hospitalisations for intentional self-harm in New Zealand, equating to an annual rate of 151.7 per 100,000 population [1]. However this figure is likely to be a considerable underestimate as a result of the way the data are collected with different hospitals having different rules about what is counted as a hospitalisation and different ways of coding self-harm. In other countries self-harm is one of the commonest reasons for presentation to the emergency department [2]. Self-harm is also important because of the link to suicide. About 1% of people who self-harm will go onto kill themselves during the next year, and a person's risk of suicide following self-harm is about 100 times that of the general population[3]. Lastly there is also a significant increase in premature mortality from other causes with up to 10% of people who present to hospital with self-harm being dead after five years with about half this premature mortality being due to physical disorders [4]. From a public health perspective, people who are admitted to hospital with intentional self-harm are an easily identifiable high-risk group, who are usually not currently in contact with health services [5], which makes this a timely opportunity for intervention particularly around suicide prevention.

### Why a complex intervention?

Current research indicates that there are several promising areas to focus on improving outcomes after self-harm.

Firstly there is regular written communication sent to patients after the episode of self-harm. In 2005 Carter and colleagues developed an intervention in which a series of eight "postcards" were sent in sealed envelopes over one year after discharge to patients who had presented at emergency departments for self-poisoning[6]. At one year follow-up patients in the intervention group had half the number of readmissions than the control group (101 versus 192) although the proportion of people re-presenting in each group was not significantly different, suggesting that any effect was attributable to reduction in multiple readmissions in a small group of patients. In a more recent trial in Christchurch by Annette Beautrais [7] of a similar intervention there was no difference in outcomes once the history of self-harm had been taken into account in both groups. However the study sample was small and included people presenting to a crisis team with suicidal ideas as well as presentations with self-harm to the emergency department.

Secondly there is problem-solving therapy (PST) which is a brief focused psychological treatment that has been shown to be significantly more effective than control conditions with regard to improvement in depression, hopelessness and problems in patients who have attended hospital after self-harm[8]. Evidence that problem solving therapy is effective in reducing repetition rates is less conclusive, although promising trends have been reported[9]. There is general agreement that problem solving therapy is a cost-effective, brief intervention that has the potential to be a feasible and effective addition to existing services[8].

Next is the issue of the high mortality rate from non suicide causes after self-harm. About 50% of the premature mortality after self-harm is due to non suicide deaths[10] and the overall rate of death may reach 15% five years after the index episode of self-harm[4]. This suggests that treating self-harm as purely a mental health problem will not address a key outcome as potentially modifiable non-mental health issues may be missed.

A related problem is the issue of patients not getting the treatment outlined in management plans made in the emergency department, perhaps because of patients' resistance to attend appointments, referrals not being made in a timely fashion or contact details recorded in the emergency department being incorrect. There is some evidence that more intensive outreach after self-harm results in better attendance in out-patients although it is unclear whether this decreases the repetition rate[9].

A difficult and controversial area is the management of risk for the individual and the team. Traditionally risk assessment in mental health, especially in the area of suicide prevention, has focused on prediction, using risk factors associated with the patient, so that patients are said to be at low, medium and high risk. The difficulty with this is that there is no evidence that clinicians can predict who will die by suicide, most people who kill themselves are low risk and most people who are high risk don't kill themselves[11]. This suggests that using risk assessment to predict who will kill themselves is flawed and that a better system is needed.

Lastly a neglected component of assessment in mental health, with the notable exception of 'cultural services", is the clinical assessment of identity and belonging in people who self-harm. (Cultural services in New Zealand are clinical services largely run by and for certain ethnic groups, in particular Maori in accordance with the Treaty of Waitangi). This is particularly surprising given that having a damaged autobiographical memory[12] and a poor sense of belonging[13] are relatively common in people who self-harm. For this reason we propose as part of the "package of care" that everybody receives a "cultural assessment" to assess their sense of belonging and for those who receive problem solving they will be prompted to consider "not belonging" as a potential problem. "Not belonging" can be operationalised as being socially isolated which can potentially be addressed by problem solving therapy.

We propose to include each of these components in a package of care delivered to individuals after they present with self-harm. By combining them together we aim to replicate the package of interventions that a clinical "self-harm team" could reasonably deliver and to test the idea that the effect of the package is more than the sum of the individual parts. The parts of the package will be sending postcards over a year; the offer of brief problem solving therapy; patient support which will be a mainly telephone based system of case management to "stop people falling through the cracks" after their presentation to hospital; vouchers that will allow patients to access their general practitioner (GP) for free with an emphasis on physical health checks and ensuring registration with a GP; a systemic approach to identifying and managing modifiable risk factors in the patient and the self-harm team based on the principles successfully used in managing risk in aviation; and a cultural assessment focused on sense of belonging.

### Why use a Zelen design?

Systematic reviews have identified small randomised trials of unrepresentative patients as a problem in this area. This study uses a double consent Zelen randomisation design. In this design individuals are randomised before they give consent. The reason for choosing this design, rather than the standard randomised controlled trial design, is that in a standard randomised controlled trial clients are required to understand complex concepts such as randomisation and clinical equipoise before giving consent so that in effect they are asked to consent to randomisation rather than a particular intervention. Such an approach is likely to be inappropriate for people in crisis, in an emergency room, and who are often physically unwell following a self-harm episode[14]. Consequently the use of a Zelen design has the potential to improve recruitment rates [15] as the conversation with eligible patients is simpler as patients are offered the choice of accepting or declining the treatment they have been offered. Also the people who consent to randomisation in conventional trials may well be unrepresentative of the people who present following self-harm [9]. More importantly, if people are offered the possibility of receiving problem-solving therapy but then find that they have been randomised to receive treatment as usual only, this may result in higher rates of non-compliance in the control group as well as the possibility of "resentful demoralisation" [16], resulting in higher drop-out rates from the control group or reporting of worse outcomes on self-completed measures.

The study aim is to investigate the effectiveness of a package of care plus treatment as usual compared to treatment as usual in people who presented to hospital after self-harm. Specifically we have the following hypotheses:

1. The package of care plus treatment as usual is more effective than treatment as usual in reducing hospital re-attendance for self-harm in the first year. This is the primary outcome.

2. The package of care plus treatment as usual is more effective than treatment as usual in reducing self-reported repetition of self-harm hopelessness, depression, anxiety and health service use at three months and one year.

3. The package of care plus treatment as usual will improve quality of life and function at three months and one year compared to treatment as usual.

4. The package of care will be more cost effective than treatment as usual.

## Methods

### Design

We will use a Zelen randomised controlled design to compare the package of care plus treatment as usual to treatment as usual in people who presented to hospital with self-harm.

### Setting

The study will be conducted in four hospitals in three District Health Boards (DHB) in New Zealand - Waitemata DHB (North Shore Hospital and Waitakere Hospital), Counties Manukau DHB (Middlemore Hospital) and Northland DHB (Whangarei Hospital). Waitemata DHB provides health services for a population of about 525,000 people in urban north and west Auckland and a rural area north of the city with about 17% of its population living in the most deprived areas; Counties Manukau provides health care for 470,000 people in the South of Auckland and serves a population that is relatively young with a high proportion of Maori and recent immigrants and about a third of the population living in areas that are very deprived (http://www.cmdhb.org.nz/about_cmdhb/overview/population-profile.htm); Northland DHB serves a mainly rural area of about 150,000 characterised by a large Maori population, widely dispersed rural communities and a disproportionately high level of socio-economic deprivation.

### Participants

Patients who present to hospital through the emergency department after self-harm will be eligible if they are not at school and are able to give informed consent. Patients who require an interpreter will be included in the study.

Potential participants will be excluded if they are aged under 17; are still at school; are unable to give informed consent to be part of the study, that is, if they are too mentally unwell (for example they are psychotic or hypomanic); if they are too physically unwell (for example, they are in a coma or with lowered level of consciousness); or if they are severely cognitively impaired.

People who identify as Maori will be recruited into Te Ira Tangata the "sister-study" to ACCESS which is a Zelen randomised controlled trial that will be offering a culturally appropriate intervention to Maori who present with self-harm. There will be times (at the start of the ACCESS trial) and places where Te Ira Tangata will not be recruiting when Maori who present with self harm will be recruited into ACCESS. This will enable us to access the impact of a Maori team versus a mainstream service on the engagement, management and outcomes of Maori who present with self-harm.

Self-harm is defined as intentional self-poisoning or self-injury, irrespective of motivation. Self-poisoning includes the intentional ingestion of more than the prescribed amount of any drug, whether or not there is evidence that the act was intended to result in death. This also includes poisoning with non-ingestible substances (for example pesticides or carpet cleaner), overdoses of 'recreational drugs' and severe alcohol intoxication where the clinical staff considers such cases to be an act of intentional self-harm. Self-injury is defined as any injury that has been intentionally self-inflicted [17].

We will assess the degree of suicidal intent by using a modified self report version of the Beck Suicide Intent Scale which we will use in the analysis to assess the impact suicidal intent on outcomes.

### Recruitment

Following a psychosocial assessment by a non-study mental health clinician, patients will be handed a card by non-study clinician which informs them that they will be approached to participate in a study about what happens after self-harm (see Additional File 1). If patients do not want to be contacted they will be asked to inform one of the non-study staff. Eligibility for the study will be assessed by a research therapist reviewing the notes. Eligible patients will be randomised and then approached by the research clinician to explain the study and to request consent to participate.

The maximum delay between the psychosocial assessment and the attempt to obtain consent is four days to allow for weekends and public holidays when research staff are not available. In practice we aim to approach potential participants within 24 hours of their presentation to the hospital. The approach will occur either within the Emergency Department, in hospital if the person is admitted, or where necessary, by telephone after the patient is discharged. An interpreter will be made available to any person who requests one.

### Randomisation and blinding

As this is a Zelen trial randomisation will be prior to obtaining consent. All eligible participants are allocated randomly to the intervention or usual care groups using a central computerised randomisation system at the Clinical Trials Research Unit (http://www.ctru.auckland.ac.nz). Stratified minimisation randomisation will be used to ensure a balance in key prognostic factors between the study groups: site (Waitemata DHB, Counties Manukau DHB, Northland DHB), history of self-harm (none, repeater), and method of self-harm (overdose, self injury, both). The assessors will be blind to the intervention group at the three and twelve months follow up assessments.

### Intervention group

People who are randomised and consent to receive the intervention will receive a six element package of care comprised of:

1. Patient support for up to two weeks. This will consist of one or two face-to-face or telephone sessions depending on patient preference and feasibility over the two week period following the participant's discharge from hospital. These sessions will involve obtaining the discharge plan developed by the assessing clinicians, checking that the patient understands it, identifying potential barriers to implementation of the plan and assisting the patient to follow through with the plan. In other words, the primary aim of patient support will be to ensure patients do not "fall through the cracks". The research clinicians will be expected to liaise with the mental health crisis and community mental health teams; alcohol and drug services; primary care and non health services. Each patient support session will include a risk assessment asking about thoughts and plans for self-harm. If a patient is identified as being at risk of self-harm the risk management protocol will be followed.

2. Postcard contact for one year. Eight postcards will be sent in sealed envelopes in months 1,2,3,4,6,8,10 and 12 after the index episode. The cards will contain a short message stating that we hope things are going well and inviting them to write us a note if they wish to (see Additional File 2). Each envelope will contain a return stamped addressed envelope.

3. Problem solving therapy. This will consist of up to four to six sessions in the four weeks after the participant's index presentation to hospital for self-harm. Research clinicians will assess the participant's eligibility for brief problem solving therapy prior to and at the initial patient support session. Patients may be ineligible for brief problem solving therapy if they are already receiving psychotherapy (for example if they are receiving Dialectical Behaviour Therapy), if brief problem solving therapy would contradict their management plan, if they live or are moving out of area, if they are in prison or if there is a risk of harm to the research clinician. The problem solving therapy we will use in the treatment package will be conducted with individual patients and is based on the model originally defined by D'Zurilla and Goldfried [18]. Problem solving therapy sessions will aim to teach the person to recognise and identify current problems and will provide them with a structured approach to problem solving. A clinician manual and a participant workbook will be used by the research team to guide the structure of problem solving therapy sessions. Sessions will be audiotaped.

4. Improved access to primary care. We will encourage participants to attend their GP for a physical health check paying particular attention to cardiovascular risk factors especially alcohol and smoking. We will use GP vouchers to facilitate these visits.

5. A risk management strategy. The teams will also pilot a risk management strategy around the management of suicidal patients. This will consist of a checklist for patient support to ensure that key tasks are completed and questions asked. Secondly the research team will meet once a week to discuss adverse events defined as repeat episodes of self-harm, hospital re presentation for any reason and suicides. A record will be kept of these discussions, including any changes to process as a result of these discussions, and circulated to the team in the form of a "risk bulletin". The research team will also receive training in crew resource management.

6. Cultural assessment. We have two aims here, the first is to increase the number of people who receive cultural services after self-harm. From our previous study we found that the input of Maori services after self-harm was very rare. While we are recruiting Maori participants into the trial (prior to Te Ira Tangata starting or at sites where Te Ira Tangata is not running), cultural services may involve liaising with Maori non government organizations and Maori mental health services. Throughout the study the cultural assessment will involve liaising with Pacific Island and Asian services if appropriate. The second aim will be to complete a cultural assessment on everyone (that is including people of European descent) paying particular attention to the sense of belonging and feelings around ethnicity. Problems with sense of belonging will be included in the problem solving checklist for patients.

### Treatment as Usual

Treatment as usual following self-harm varies and may involve referrals to multi-disciplinary teams for psychiatric or psychological intervention, referrals to crisis teams and/or recommendations for engagement with community alcohol and drug treatment centres. The discharge plan may include referrals to more than one health care provider, or may consist solely of referral back to the patients' General Practitioner.

#### Treatment as usual assessment

Treatment as usual for all participants will be assessed by self report using a written questionnaire and telephone interview by a research assistant blind to treatment allocation; a review of DHB records; and by using the National Minimum Dataset from the Ministry of Health Information Directory to record hospital contacts and contact with mental health services. The National Minimum Dataset contains routinely collected information on all hospital discharges in New Zealand linked to a patient's individual National Health Index number.

### Outcome measures (Table 1)

**Table 1:** Outcome measures

Outcome measure	Description	Explanation	Administered
**Primary**			

Hospital repetition of self-harm		Data on hospital contacts from participating DHB's and the New Zealand Health Information Service National Minimum Dataset	Three and twelve months

**Secondary**			

Hopelessness	Beck Hopelessness Scale (BHS)[22]	Best predictor of subsequent self-harm. Scores on a range of 0 to 20 with higher scores indicating greater hopelessness.	Baseline, three and twelve months

Depression and anxiety	Hospital Anxiety and Depression Scale (HADS)[23]	Self report scale. Scores of 10 and above on the anxiety and depression sub scales indicate clinically significant symptoms.	Baseline, three and twelve months.

Health status	EQ-5D [24]	A generic health-related quality of life index that can be related to costs.	Baseline, three months and twelve months

Self report repetition of self-harm		Self report assessed by telephone interviewer blind to allocation	Three and twelve months

Social functioning	SF-36 [25]	A generic measure of functional health and well being	Baseline, three and twelve months

Sense of belonging	Sense of belonging instrument (SOBI) [26]	Self report scale on sense of belonging to a community and ethnicity	Baseline, three and twelve months

Seriousness of suicide attempt	Self rated objective part of the Beck Suicide Intent Scale (BSIS)[27]	Self report scale indicating the degree of suicidal intent of the self-harm episode	Baseline

Costs following index attempt	Health service use, costs of attending care, cost of medication and time off work	Self report assessed by telephone interviewer blind to allocation	Three and twelve months

The primary outcome measure is hospital repetition for self-harm during the year after the index presentation.

The secondary outcome measures are:

1. Self reported repetition for self-harm assessed at three months and one year.

2. Hopelessness measured by the Beck Hopelessness Scale at baseline, three months and one year.

3. Depression and anxiety measured by the Hospital Anxiety and Depression Scale at baseline, three months and one year.

4. Overall mortality (that is suicide deaths plus other causes of death) and suicide deaths at three months, one year, five years and ten years.

5. Costs after the index presentation including health service use, costs of attending care, cost of medication and time off work at three months and one year.

6. Quality of life and social function as measured by the EQ-5D (http://www.euroqol.org/) and the SF36 at baseline, three months and one year.

Outcome measures will be collected in several different ways.

#### Self rating

Rating scales will be by self rating.

#### DHB records

We will inspect these for episodes of repetition and health service use including face to face contact with mental health services and hospital admissions.

***Ministry of Health Information Directorate National Minimum Dataset ***(http://www.moh.govt.nz/moh.nsf/indexmh/dataandstatistics-collections-nmds) ***and Mortality Collection ***(http://www.moh.govt.nz/moh.nsf/indexmh/dataandstatistics-collections-mortality) We will inspect these for health service use (both general and mental health), hospital representation for self-harm and for mortality data. (It is necessary to look at national data on these measures as we found in our previous trial that at least 50% of people who self-harm change address over twelve months often outside the DHB where they presented).

#### Self report by structured interview

Information for the economic analysis and self report of repetition will be gathered by telephone interview at three months and one year after the index attempt (see Additional File 3 for pro forma). Telephone interviewers will be blind to the allocation of subjects. Blinding will be tested by asking the interviewers to nominate which group the subject was enrolled in. The economic analysis will also use a brief measure of quality of life the EQ-5D and interviewers will ask about health service use; costs associated with this; time off work; time off work for family to care for the participant; changes in benefit; changes in occupation; and drug use and cost. Participants will have the option of completing these measures in a face to face interview if, for example, they do not have a phone.

### Process evaluation

A process evaluation will explore the implementation, receipt and context of the intervention with a view to helping understand the results in accordance with the Medical Research Council's guidelines[19] on assessing complex interventions. This will describe the processes in the intervention and control groups, provide information about the contexts in which the treatments are delivered and supply information about the experience of being part of the trial. The process evaluation is described in Table 2 below. The self-harm teams will also receive weekly supervision, the main themes of which we will incorporate into the process evaluation.

**Table 2:** Process evaluation in ACCESS

Data collection method	Data collected
Programme documentation and observation (to assess fidelity, dose and reach)	Number of sessions of patient support Number of PST sessions Completion of PST Audiotaping of PST sessions Examination of written client PST research records Number of clients where sense of belonging addressed in PST Use of GP voucher Number of postcards sent Summary of discussions around adverse events Proportion of clients who received the interventions in each centre

Structured interviews (to assess barriers, facilitators and suggestions for improvement)	Interview research clinicians re barriers and facilitators to the interventions plus suggestions for improvement Interview purposive sample of patients re what helped and what did not help plus suggestions for improvement Twelve month telephone interview of all patients what helped and what did not help Interview GP's who saw clients through GP voucher to assess their perception of the intervention and suggestions for improvement Interview focus group of staff in mental health services re barriers and facilitators to interventions plus suggestions for improvement

### Process evaluation analysis

Numerical data will be entered into the Clinical Trials Research Unit web based data entry system specifically designed for this study. Information from the examination of patient notes and audiotapes will be used to assess the adherence of therapists to the manual in a 10% random sample of those who completed problem solving therapy. Data from structured interviews and focus groups will be analysed for emergent themes using NVIVO.

## Statistical methods

### Power analysis

We know from our previous study that people who agree to receive problem solving therapy have a hospital repetition rate at one year of about 13%. The rate of repetition in people who receive usual care is about 20% - that is a relative risk reduction of 35% which would be clinically important. To demonstrate that such a difference is unlikely to be due to chance at a significance level of 0.05 with 80% power we will need to recruit 440 people into each arm of the trial. In the two Auckland DHBs (Waitemata and Counties Manakau) and Northland conservatively 1450 people present with self-harm a year [20]. Reducing this figure by 15% (which is the proportion who would be under 17) would mean a potential pool of about 1200 eligible people a year of whom 600 (50%) would agree to be in a trial, so 900 in 18 months. This number will also have enough power to detect a 50% reduction in overall mortality at 5 and 10 years (from 10% to 5%). We will apply for further funding to allow follow up of this cohort.

### Analysis

Statistical analysis will be by the biostatistics team of the Auckland Clinical Trials Research Unit (CTRU).

Data from the trial will be entered into an Oracle database at the CTRU and extracted into SAS for analysis. All statistical analyses will be performed using SAS version 9.2 (SAS Institute Inc. Cary NC). All statistical tests will be two-tailed and a 5% significance level maintained throughout the analyses. Assessment of baseline comparability of the intervention and control group will be carried out via descriptive analyses for demographic information, method of self-harm and previous history of self-harm. The proportion of people repeating self-harm in each group will be analysed using chi-squared test. The number of self-harm re-presentation episodes for the hospital and self report outcomes during follow-up will be analysed using negative binomial regression. Kaplan-Meier curves and Cox proportional hazards regression modeling will be used to analyze time to first re-presentation to hospital for self-harm and time to event for the mortality outcomes. The change from baseline to 3 months and one year in each of the repeated continuous outcomes will be analysed using mixed model regression. If baseline characteristics are found to be substantially different between the groups we will adjust for these in the regression modeling. All analyses will be conducted on patients who are randomised and consented except for the re-presentation to hospital and time to re-presentation outcomes which will be an intention to treat analysis that includes all randomised patients including those that did not consent. (This is because information on these outcomes are routinely collected in New Zealand as part of the National Minimum Dataset). We plan to analyse those with and without a history of self harm at the index episode together and separately.

In addition sensitivity analyses will be conducted using a CACE analysis[21] where appropriate which takes into account the fact that after randomisation not everyone in a Zelen design agrees to take part in the study. This has the effect of diluting any treatment effect and introducing a possible self selection bias. A CACE analysis is an attempt to correct for this.

### Cost effectiveness analysis

We aim to collect the following data from all patients in the trial at three months and one year after the date of their index attempt. The data will be collected by a research assistant by telephone interview with the patients, by examination of routinely collected health data and liaison with the finance departments of the relevant health care providers.

#### Costs to patients

• Time off work

• Distance travelled for treatment for all disorders

• Time taken for treatment

• Costs for attending general practice - travel, payment to general practice, time off work

• Costs of family to attend treatment or provide support for the patient (for example taking time off work to be with the patient)

• Cost of medication

• Benefits claimed

#### Costs to health care provider

• Staff salaries for providing treatment including the problem solving therapy (therapist and in-patient treatment)

• Length of stay in hospital

• Cost of treatments - for example care in intensive care or burns unit, cost of medication

• Overheads

##### Analysis

All analyses will be carried out on an intention-to-treat basis for total costs over three months and one year. We will perform multiple regression to adjust for baseline characteristics including age, sex, number of previous attempts and Beck Hopelessness Score. We intend to perform an incremental analysis of costs and consequences using the primary outcome measure as the number of repetitions averted. From this we will produce cost-effectiveness acceptability curves for the intervention.

We also intend to perform a sensitivity analysis to test how the costs and consequences of self-harm change within a range of costs for the different economic inputs. We anticipate that the model will be most sensitive to changes in the costs of in-patient medical care. We will also test the sensitivity of the results to productivity losses and costs of mental health treatment.

### Termination of the study

Termination will be considered if there is 10% absolute greater number of adverse events (re-presentations to hospital for self-harm) in the treatment group than in the usual care group at three months. Suicides will be reported to the relevant ethics committee. Unblinded analyses to assess excess harm will be conducted at one year by an independent statistician at CTRU (not the study statistician). One year was chosen as any shorter time will mean that there will be few outcomes and any longer time is close to the end of the intervention at 18 months. For this analysis the Haybittle-Peto stopping boundary will be used which is based on a three standard deviation rule corresponding to a two sided test P = 0.003 stopping rule. This does not affect the power calculation of the study.

## Discussion

The study, due to report its findings in 2012, tests the effectiveness of a complex package of interventions in the management of people who present to hospital with intentional self-harm. It uses a novel design to try and overcome the problems of previous trials which have recruited small numbers of unrepresentative people. A strength of the study is that it is a pragmatic trial which aims to recruit large numbers and does not exclude people if English is not their first language. The analysis of the primary outcome is a true intention to treat analysis of everyone randomised not just those who agree to the intervention. This provides greater information about the impact of the intervention when introduced into a service compared to a standard randomised controlled trial. A potential limitation is the analysis of the results which is complex and may underestimate any effect on the secondary outcomes if a large number of people refuse their consent in the group randomised to problem solving therapy as they will effectively cross over to the treatment as usual group.

## Abbreviations

BHS: Beck Hopelessness Scale; BSIS: Beck Suicide Intent Scale; CACE: Complier Average Causal Effect; CTRU: Clinical Trials Research Unit; DHB: District Health Board; GP: General Practitioner; HADS: Hospital Anxiety and Depression Scale; PST: Problem solving therapy; SF36: Short Form (36) Health Survey; SOBI: Sense of belonging instrument;

## Competing interests

The authors declare that they have no competing interests.

## Authors' contributions

All authors contributed to the study design and study protocol. SH is the principle investigator, CS is the project manager and NC is the project co-ordinator. SH and CS drafted the article. All authors have read and approved the final manuscript.

## Supplementary Material

Additional file 1 Card letting people know they will be contacted. Click here for file

Additional file 2 Postcard. Click here for file

Additional file 3 Follow up telephone interview proforma. Click here for file

## References

[B1] Ministry of Health Suicide Facts: 2005-2006 data 2007 Wellington: Ministry of Health

[B2] Hawton K Fagg J Simkin S Bale E Bond A Trends in deliberate self-harm in Oxford, 1985-1995. Implications for clinical services and the prevention of suicide British Journal of Psychiatry 1997 171 556 560 10.1192/bjp.171.6.556 9519096

[B3] Hawton K Fagg J Suicide, and other causes of death, following attempted suicide British Journal of Psychiatry 1988 152 359 366 10.1192/bjp.152.3.359 3167371

[B4] Ostamo A Lonnqvist J Excess mortality of suicide attempters Social Psychiatry & Psychiatric Epidemiology 2001 36 1 29 10.1007/s001270050287 11320805

[B5] Suominen K Isometsa E Ostamo A Lonnqvist J Health care contacts before and after attempted suicide Social Psychiatry & Psychiatric Epidemiology 2002 37 2 89 94 10.1007/s127-002-8220-y 11931093

[B6] Carter GL Clover K Whyte IM Dawson AH D'Este C Postcards from the EDge project: randomised controlled trial of an intervention using postcards to reduce repetition of hospital treated deliberate self poisoning BMJ 2005 331 7520 805 10.1136/bmj.38579.455266.E0 16183654 PMC1246077

[B7] Beautrais AL Gibb SJ Faulkner A Fergusson DM Mulder RT Postcard intervention for repeat self-harm: randomised controlled trial The British Journal of Psychiatry 2010 197 1 55 60 10.1192/bjp.bp.109.075754 20592434

[B8] Townsend E Hawton K Altman DG Arensman E Gunnell D Hazell P House A Van Heeringen K The efficacy of problem-solving treatments after deliberate self-harm: meta-analysis of randomized controlled trials with respect to depression, hopelessness and improvement in problems Psychological Medicine 2001 31 6 979 988 11513383 10.1017/s0033291701004238

[B9] Hawton K Townsend E Arensman E Gunnell D Hazell P House A van Heeringen K Psychosocial versus pharmacological treatments for deliberate self harm Cochrane Database of Systematic Reviews 2000 2 CD001764 10.1002/14651858.CD001764 10796818

[B10] Hawton K Harriss L Zahl D Deaths from all causes in a long-term follow-up study of 11,583 deliberate self-harm patients Psychological Medicine 2006 36 3 397 405 10.1017/S0033291705006914 16403244

[B11] Appleby L Shaw J Amos T McDonnell R Harris C McCann K Kiernan K Davies S Bickley H Parsons R Suicide within 12 months of contact with mental health services: national clinical survey Bmj 1999 318 7193 1235 1239 10231250 10.1136/bmj.318.7193.1235 PMC27859

[B12] Pollock LR Williams JM Effective problem solving in suicide attempters depends on specific autobiographical recall Suicide and Life Threatening Behavior 2001 31 4 386 396 10.1521/suli.31.4.386.22041 11775714

[B13] Conner KR Britton PC Sworts LM Joiner TE Suicide attempts among individuals with opiate dependence: The critical role of belonging Addictive Behaviors 2007 32 1395 1404 10.1016/j.addbeh.2006.09.012 17097813

[B14] Hatcher S Sharon C Coggan C Beyond randomized controlled trials in attempted suicide research Suicide & Life-Threatening Behavior 2009 39 4 396 407 10.1521/suli.2009.39.4.396 19792981

[B15] Andrew M Vegh P Caco C Kirpalani H Jefferies A Ohlsson A Watts J Saigal S Milner R Wang E A randomized, controlled trial of platelet transfusions in thrombocytopenic premature infants Journal of Pediatrics 1993 123 2 285 291 10.1016/S0022-3476(05)81705-6 8345429

[B16] Adamson J Cockayne S Puffer S Torgerson DJ Review of randomised trials using the post-randomised consent (Zelen's) design Contemporary Clinical Trials 2006 27 4 305 319 10.1016/j.cct.2005.11.003 16455306

[B17] Hawton K Bergen H Casey D Simkin S Palmer B Cooper J Kapur N Horrocks J House A Lilley R Self-harm in England: a tale of three cities. Multicentre study of self-harm Social Psychiatry & Psychiatric Epidemiology 2007 42 7 513 521 10.1007/s00127-007-0199-7 17516016

[B18] D'Zurilla TJ Goldfried MR Problem solving and behaviour modification Journal of Abnormal Psychology 1971 78 107 126 4938262 10.1037/h0031360

[B19] Craig P Dieppe P Macintyre S Michie S Nazareth I Petticrew M Developing and evaluating complex interventions: new guidance 2008 Medical research Council

[B20] Hatcher S Sharon C Collins N Epidemiology of intentional self-harm presenting to four district health boards in New Zealand over 12 months, and comparison with official data Australian and New Zealand Journal of Psychiatry 2009 43 7 659 665 10.1080/00048670902970833 19530023

[B21] Hewitt CE Torgerson D Miles JNV Is there another way to take account of non-compliance in randomized controlled trials? CMAJ 2006 175 347 348 16908892 10.1503/cmaj.051625 PMC1534095

[B22] Beck AT Weissman A Lester D Trexler L The measurement of pessimism: the Hopelessness Scale Journal of Consulting & Clinical Psychology 1974 42 6 861 865 4436473 10.1037/h0037562

[B23] Zigmond A Snaith R The hospital anxiety and depression scale Acta Psychiatrica Scandinavica 1983 67 6 361 370 10.1111/j.1600-0447.1983.tb09716.x 6880820

[B24] Rabin R de Charro F EQ-5D: a measure of health status from the EuroQol Group Annals of Internal Medicine 2001 33 337 343 10.3109/07853890109002087 11491192

[B25] Ware JE Sherbourne CD The MOS 36-item short-form health survey (SF-36). I. Conceptual framework and item selection Mdical Care 1992 30 6 473 483 10.1097/00005650-199206000-00002 1593914

[B26] Hagerty BM Patusky K Developing a measure of sense of belonging Nursing Research 1995 44 1 9 13 7862549

[B27] Beck AT Schuyler D Herman I Beck AT, Resnick HLP, Lettieri DJ Development of suicidal intent scales The Prediction of Suicide 1974 Philadelphia: Charles Press

