# Supplementary figures and images for: The ACCESS study a Zelen randomised controlled trial of a treatment package including problem solving therapy compared to treatment as usual in people who present to hospital after self-harm: study protocol for a randomised controlled trial
Source: Trials. 2011 May 26;12:135. doi: 10.1186/1745-6215-12-135 (PMC3117717; doi:10.1186/1745-6215-12-135)

Additional file 1: Card letting people know they will be contacted


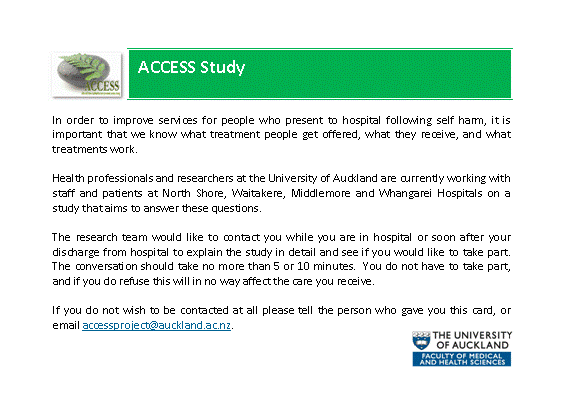

Supplement: Additional file 1 — Card letting people know they will be contacted. [file 1745-6215-12-135-S1.DOC]

Additional file 2: Postcard


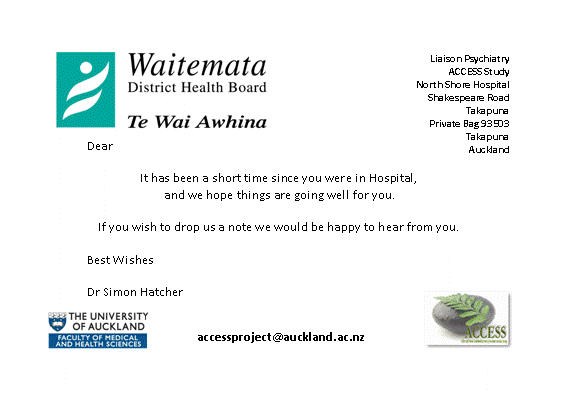

Supplement: Additional file 2 — Postcard. [file 1745-6215-12-135-S2.DOC]
